# Adherence to a Mindfulness and Relaxation Self-Care App for Cancer Patients: Mixed-Methods Feasibility Study

**DOI:** 10.2196/11271

**Published:** 2018-12-06

**Authors:** Michael Mikolasek, Claudia M Witt, Jürgen Barth

**Affiliations:** 1 Institute for Complementary and Integrative Medicine University Hospital Zurich and University of Zurich Zurich Switzerland; 2 Institute for Social Medicine, Epidemiology and Health Economics Charité – Universitätsmedizin Berlin Berlin Germany; 3 Center for Integrative Medicine University of Maryland School of Medicine Baltimore, MD United States

**Keywords:** mobile app, mindfulness, relaxation, cancer, patient compliance

## Abstract

**Background:**

Cancer is highly prevalent worldwide and can cause high levels of distress in patients, which is often neglected in medical care. Smartphone apps are readily available and therefore seem promising to deliver distress-reducing interventions such as mindfulness and relaxation programs.

**Objective:**

This study aimed to evaluate the feasibility of a mindfulness and relaxation app for cancer patients. We looked at characteristics of participating patients in a mobile health (mHealth) study, including adherence to the app intervention, predictors for adherence, and patients’ feedback regarding the app.

**Methods:**

In this prospective observational study with a mixed-methods approach, cancer patients received a mindfulness and relaxation self-care app. Cancer patients were recruited online and through hospitals in Switzerland. We assessed self-reported measures (eg, quality of life, anxiety, depressive symptoms, openness to experience, resistance to change) at baseline, and the app gathered data on patients’ practicing time. With 8 semistructured interviews, we obtained patients’ feedback about the app and recommendations for improvements. We looked at 3 dimensions of the Reach, Effectiveness, Adoption, Implementation, and Maintenance framework (reach, adoption, and maintenance) and analyzed data for adherence for the first 10 weeks of the app intervention. We report descriptive statistics for patient characteristics and app use. For the prediction of adherence, we used Kaplan-Meier analyses with log-rank tests and a Cox proportional hazards regression.

**Results:**

Data from 100 cancer patients (74 female) showed that 54 patients were using the app exercises continuously until week 10. In continuous app users, the median number of exercises per week dropped from 4 (interquartile range, IQR 1-7) at week 1 to a median of 2 (IQR 1-4) at week 10. Our analyses revealed 4 significant predictors for better adherence: female gender, higher openness to experience, higher resistance to change, and more depressive symptoms. Interviews revealed that the patients generally were satisfied with the app but also made suggestions on how to improve it.

**Conclusions:**

Our study indicates that a mindfulness and relaxation mHealth intervention for cancer patients is feasible with acceptable adherence and largely positive feedback from patients.

**Trial Registration:**

German Clinical Trials Register DRKS00010481; https://www.drks.de/drks_web/navigate.do?navigation Id=trial.HTML&TRIAL_ID=DRKS00010481 (Archived by WebCite at http://www.webcitation.org/73xGE1B0P)

## Introduction

### Background

Cancer is highly prevalent worldwide, with an estimated 14 million newly diagnosed patients per year [1]. According to the World Health Organization, cancer is the second leading cause of death, with an increasing economic impact over recent years [2]. For patients, the diagnosis of cancer and subsequent treatment (eg, radiation or chemotherapy) can cause high levels of distress [3,4]. About every second cancer patient has clinically relevant distress, with elevated levels of depression or anxiety [5]. However, psychological support of patients is often not implemented in standard medical care [6-8]. In addition, many patients neglect their distress and do not seek psychosocial support [9]. However, untreated distress can reduce quality of life as well as lower adherence with recommended medical care and, therefore, negatively affect patients’ recovery [8,10]. Thus, a variety of treatments such as counseling and Mind Body Medicine (MBM) interventions have been suggested to reduce cancer patients’ distress during initial care and rehabilitation [11-13].

MBM focuses on the interactions between psychological and biological processes and their impact on health [14,15] and has shown beneficial effects in reducing cancer patients’ distress [13,16]. MBM usually combines a variety of interventions, such as exercise, Qigong, relaxation, and mindfulness meditation [14]. Some of these interventions, for example, mindfulness and relaxation, are also commonly used on their own and have been studied extensively with promising effects in both healthy and patient populations [15,17-20]. In addition, an increasing number of cancer patients are interested in or use mindfulness or relaxation interventions [21].

Regular practice is crucial for the effect of mindfulness and relaxation-based interventions, which can be difficult to achieve due to lack of motivation, time constraints, as well as limited access to interventions [22]. Further restrictions for regular practice and access-limiting factors include geographical distance, financial constraints, lack of treatment providers or lack of knowledge thereof [8,9,23]. For cancer patients, regular practice might additionally be hindered due to restrictions caused by cancer (eg, fatigue and nausea) and its comprehensive treatments.

Mobile health (mHealth) interventions might overcome some of the restrictions of face-to-face interventions. The access to interventions can be easier due to a large and increasing number of smartphone owners [24]. In 2017, more than 32% of the world population and more than 60% of the population in Western Europe and North America owned a smartphone [25]. In addition, mHealth interventions have some specific advantages compared with face-to-face interventions. These advantages include easy and pervasive access to information (ie, psychoeducation), engaging audio and/or visual material, potential customization of the app according to client’s preferences and needs, provision of regular feedback, reminders, and reduced perceived stigmatization due to potentially less therapist contact [24,26]. mHealth interventions can also be a good support for patients’ self-care [26]. Such self-care interventions can have beneficial effects on cancer patients’ distress and quality of life [27] and can be implemented via an app using audio instructions.

To date, mHealth interventions using a mindfulness or relaxation intervention strategy have been under-researched, with the focus of studies primarily on Web-based electronic Health (eHealth) interventions [28]. For eHealth interventions, studies indicate that mindfulness- and relaxation-based interventions can have beneficial effects on health outcomes in various populations, including cancer patients [28-30]. Beneficial effects of eHealth were reported for stress, well-being, anxiety, depression, and mindfulness. The majority of available primary studies in these reviews focused on eHealth interventions, with a partial emphasis on Web-based patient-therapist interactions. However, less is known about the feasibility and effectiveness of mHealth interventions, and certain disadvantages (eg, technical problems, concerns about data security) are well known [26]. Eysenbach [31] coined the term “law of attrition,” which emphasizes that early and rapid attrition rates are an inherent problem in technology-delivered interventions. Especially in self-care interventions with regular exercises, good adherence itself often becomes an intervention goal. Although recent eHealth studies report acceptable rates of adherence (eg, 60% completed 4 or more out of 6 modules [32] and 71% practiced more than 50% of the days during 8 weeks [33]), little is known about the adherence to mindfulness and relaxation mHealth programs for cancer patients. Therefore, when setting up a self-care mHealth intervention, it is important to know which factors might influence the patient adherence.

### Objective

The aim of this mHealth study, then, was to evaluate the feasibility of a mindfulness and relaxation app for cancer patients and its impact on health outcomes according to the Reach, Effectiveness, Adoption, Implementation, and Maintenance (RE-AIM) framework [34]. In this analysis, we looked at the characteristics of patients who participated in this mHealth study, adherence and predictors for adherence, as well as patients’ feedback regarding the mHealth intervention from interviews.

## Methods

### Study Design

We performed a prospective observational study using a mixed-methods approach. Quantitative data consisted of 4 paper-and-pencil questionnaires sent to cancer patients at baseline, weeks 4, 10, and 20. Qualitative data consisted of semistructured interviews with 8 cancer patients. Corresponding to the principles of theoretical sampling [35], we recruited the interviewees based on the sample distributions of gender and intervention dropouts versus continuous app users. We conducted individual qualitative interviews over the telephone with these patients; selecting 4 of them who used the app on a regular basis and 4 who did not use the app regularly. We conducted qualitative interviews with these patients; 4 of them used the app on a regular basis and 4 did not use the app regularly. This study was guided by the RE-AIM evaluation framework [34], which consists of the following 5 dimensions: reach, effectiveness, adoption, implementation, and maintenance. For this analysis, we focused on 3 dimensions, namely reach, adoption, and maintenance during the first 10 weeks of the intervention. The dimensions effectiveness and implementation as well as results about the entire 20 weeks will be reported in an upcoming paper. The cantonal ethics committee granted ethical approval for the study (BASEC-Nr. 2016-00258) in April 2016, and the study was positively audited within the regular ICH-GCP audit of the University Hospital Zurich in August 2016. We registered the study in the German Clinical Trials Register (DRKS00010481).

### Eligibility Criteria

We included female and male cancer patients (18 years or older) with any cancer diagnosis at any stage of cancer, who owned either an iOS- or Android-based smartphone with at least a weekly connection to the internet. We excluded patients if they had suicidal ideation or insufficient German language skills. Furthermore, patients who intended to move to another country and patients with insufficient knowledge on how to use a smartphone were excluded.

### Recruitment

We recruited cancer patients in 2 different settings: (1) cancer patients who participated in a supportive MBM treatment (either as individual session or as a 10-week group treatment) or (2) cancer patients without an MBM treatment.

For setting 1, cancer patients were recruited at the Institute for Complementary and Integrative Medicine, University Hospital Zurich (ICI). All available cancer patients in an MBM group treatment (between June 2016 and December 2017; 12 groups with a total of 81 patients) were invited at the third session of the course to participate in the study. Therefore, enrolled patients from setting 1 were using the app partially in parallel to the MBM group treatment. In addition, we asked the health professionals of the ICI to distribute leaflets during individual MBM consultations with cancer patients.

For setting 2, patients were recruited through the University Hospital Zurich (ICI, cancer center, Department of Radiotherapy, and University Hospital Facebook page), University Hospital Basel, and the Cantonal Hospital Aarau. Cancer patients were informed of the study using leaflets in the waiting areas or during consultations. In addition, we informed cancer patients through the Swiss Cancer League via leaflets and their Facebook page, as well as through the Cancer League of Zurich.

Interested patients initially contacted a researcher at the ICI by phone or email and made an appointment for a 10-min telephone screening interview. During the screening interview, the researcher explained the study and assessed the eligibility of the patient. In addition, the researcher recommended that the patient carry out 1 of the 3 exercises of the app at least once a day on 5 different days per week during the 20-week intervention. However, the researcher also stated that patients were free to choose when, where, and how often they practiced. After the researcher provided all information and if the patient met the eligibility criteria, the researcher asked for contact details of the patient. Subsequently, the patient received the written study information with the informed consent form, as well as the first questionnaire by mail. We sent an email to every included patient with a code to activate the app. Thereafter, patients were able to use the app free of charge. The date of the code distribution was considered as the start of the intervention for each patient. No other verbal contact between the researcher and the patients took place after inclusion of patients.

### Intervention

The mindfulness and relaxation app comprised 2 main features: (1) mindfulness and relaxation exercises guided by audio instructions and (2) a notification feature. The first feature of the app contained 3 exercises and was the main component of the app. The exercises were mindfulness meditation, guided imagery, and progressive muscle relaxation audio files with a duration of about 15 min each. Every exercise was guided by a narrator with either a male or female voice.

The second feature of the app was a notification feature, which reminded the patient to practice daily. The patient could set the time of notification according to individual preferences. The reminder to practice popped up as a push notification on the mobile device every day at the time set by the patient. The concept of the app built on previously developed relaxation study apps of an affiliated group [36], which were designed for patients with chronic low back pain (Relaxback) and chronic neck pain (Relaxneck).The app was developed by the software company Smart Mobile Factory GmbH (Berlin, Germany). After thorough testing, the app was released in June 2016 on the Apple iTunes Store and on the Google Play Store for Android devices. After the release, the content of the app was not changed. Screenshots of the app are available in Multimedia Appendices 1 and 2.

### Outcomes

#### Reach

For the dimension reach, we looked into which and how many cancer patients participated in the study. We present baseline characteristics to describe participating patients: type of cancer, status of cancer treatment, sociodemographic data (gender, age, and highest education), distress (Distress-Thermometer [37,38]), quality of life (FACT-G, Functional Assessment of Cancer Therapy-General [39]), and anxiety and depression (HADS, Hospital Anxiety Depression Scale [40]).

The Distress-Thermometer consists of 1 item with a scale from 0 to 10 and assesses experienced distress in the past week. A score between 5 or higher is considered as clinically relevant distress [41]. The FACT-G consists of 27 items, which assess the 4 subscales: physical well-being (Cronbach alpha=.851), social well-being (Cronbach alpha=.760), emotional well-being (Cronbach alpha=.702), and functional well-being (Cronbach alpha=.794). Each item is rated on a 5-point scale (0-4), resulting in a score range of 0 to 108, with a higher score indicating a better quality of life. The HADS consists of 14 items, with 7 items for each subscale, that is, anxiety (Cronbach alpha=.787) and depression (Cronbach alpha=.667). Each item is rated on a 4-point scale (0-3), leading to a maximum score of 21 for each subscale. A score between 0 and 7 is considered normal, whereas a score between 8 and 11 is considered as borderline, and a score above 11 as caseness.

#### Adoption

For the dimension adoption, we looked at indicators of patients’ adoption of the app intervention into their regular life, adherence, and information about barriers and facilitators for regular use. For this purpose, we analyzed the use of the app during the first 10 weeks. We derived app use data from tracking the practicing time with the audios (start and end time and type of exercise used). This information was visible only for the research team (as an XML log file through the backend) and was not displayed to users. In addition, we analyzed results from interviews with patients regarding their adoption of the app intervention.

As a first indicator for app intervention adoption, we report the number of completed app exercises per week. We considered an exercise as completed if the patient used the exercise for at least 10 min (out of 15 min). As a second indicator, we report the number of intervention dropouts versus number of continuous app users per week. Intervention dropouts were defined as enrolled patients who never completed an exercise or did not complete an exercise during 4 consecutive weeks after initial practice. A patient counted as an intervention dropout in the first of the 4 weeks, in which he or she did not complete any exercise. According to our definition, a patient who never completed an exercise is an intervention dropout at week 1. Patients not classified as intervention dropouts were defined as continuous app users. Consequently, continuous app use was defined as at least weekly use of 1 or more app exercises. We also report results from 8 semistructured patient interviews, in which we inquired about patients’ general impression regarding the app, app usage, and suggestions for improvements (for interview guideline, see Multimedia Appendix 3).

#### Maintenance

For the dimension maintenance, we looked into predictors for continuous app use. First, we assumed that patients with higher openness to experience are more often continuous app users. Second, we assumed that patients with higher resistance to change are less often continuous app users. In addition, we tested in explorative analyses if quality of life (FACT-G), anxiety (HADS anxiety), depression (HADS depression) at baseline and sociodemographic data (gender and age), as well as setting are associated with continuous app use. During the interviews, we also explored possible reasons for continuous app use and intervention dropout.

We measured openness to experience with the respective subscale of the NEO 5-Factor Inventory (NEO-FFI [42]) using the 5-item short version (Cronbach alpha=.755). Each item is rated on a 4-point scale (0-4), leading to a score with a range from 0 to 20. A higher score indicates greater openness to experience. We also used the Resistance to Change (RTC) Scale [43], which consists of 17 items (Cronbach alpha=.839). Each item is rated on a 6-point scale (1-6), resulting in a score with a range from 17 to 102. A higher score indicates greater resistance to change.

### Sample Size

In this feasibility study, the sample size is an outcome in itself (ie, dimension reach in the evaluation framework). Therefore, we did not perform an a priori sample size calculation, but the aim was to recruit about 100 patients to conduct explorative analyses about the feasibility of the app with sufficient precision.

### Data Analysis

#### Quantitative Data

Trained researchers entered data from printed case report forms using REDCap electronic data capture tools [44] hosted at the University Hospital Zurich. Analyses were conducted using SPSS version 25.0 (IBM Corp, Armonk, NY, USA) [45].

For the reach analyses, we used descriptive statistics (frequencies and percentages for categorical and dichotomous variables, mean and SD for continuous variables) for baseline data on sociodemographic characteristics (gender, age, and education), health status (type of cancer, status of cancer treatment, FACT-G, HADS, and Distress-Thermometer), and the setting of the enrolled patients. For the adoption analyses, we used boxplots to report median and interquartile range (IQR) of the number of completed exercises per week (week 1 to 10) for all enrolled patients, as well as for continuous app users during the 10-week intervention. In addition, we used a Kaplan-Meier plot to visualize the number of dropouts per week. For the maintenance analyses, we used Kaplan-Meier analyses with a log-rank test to compare continuous app users (ie, reversed rate of attrition) according to different baseline variables. As predictors, we used the following categorical variables: gender, setting, age groups (18-40, 41-55, 56+), high versus low well-being (FACT-G median split at 76.83), high versus low openness to experience (NEO-FFI-O median split at 17.00), high versus low resistance to change (RTC median split at 51.00), normal versus suggestive or higher anxiety or depression (HADS anxiety or depression scores of 0-7 vs 8 or higher). Subsequently, we performed a Cox proportional hazards regression with all significant predictors in the log-rank test in the Kaplan-Meier analyses.

For missing data, we used multiple imputation to conduct the Cox proportional hazards regression with a full dataset. We carried out imputations for the sum scores of FACT-G, as missing single items are already considered in the calculation of FACT-G sum scores (FACT-G sum scores are not calculated if there are more than 50% items missing in a subscale). For HADS, NEO-FFI-O, and RTC, we imputed all items with 1 or more missing values. For all other analyses (ie, descriptive analyses for the dimensions reach and adoption, Kaplan-Meier analyses for the dimension maintenance), we used complete datasets.

#### Qualitative Data

For the interview analyses about the adoption and maintenance of the intervention, we transcribed the recorded interviews verbatim and used thematic coding for structuring the interviews using MAXQDA 11 (VERBI Software, Berlin, Germany). Thereafter, we used content analysis according to Mayring [46].

## Results

### Reach

**Figure 1:**
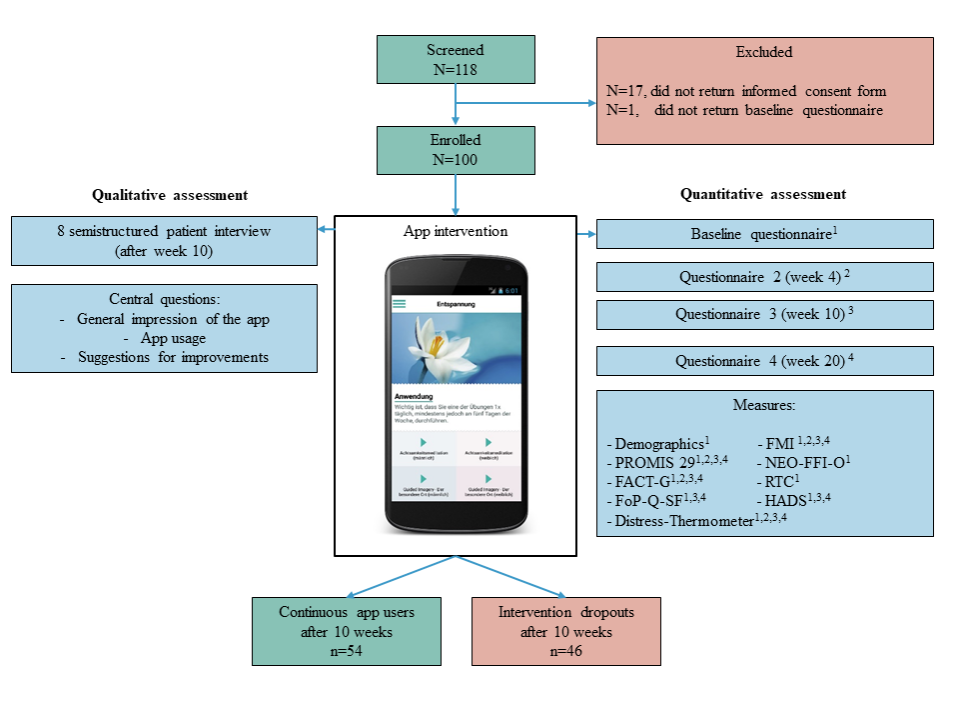
Patient flowchart. FMI: Freiburg Mindfulness Inventory; PROMIS 29: Patient Reported Outcomes Measurement Information System 29; NEO-FFI-O: NEO Five-Factor Inventory - openness to experience; FACT-G: Functional Assessment of Cancer Therapy-General; RTC: Resistance to Change; FoP-Q-SF: Fear of Progression Questionnaire - Short Form; HADS: Hospital Anxiety Depression Scale.

During the recruitment phase between June 2016 and December 2017, a total of 118 patients expressed interest in participating in the study and were screened for eligibility. All of the 118 patients fulfilled the eligibility criteria and received the informed consent form. By the end of December 2017, 100 patients signed and returned the informed consent form and were enrolled in the study (see Figure 1).

Baseline characteristics of all enrolled patients (N=100), as well as of continuous app users (54/100, 54%) and intervention dropouts (46/100, 46%) are presented in Table 1. The majority of patients (83/100, 83%) were recruited independent of the MBM treatment (setting 2). Patients were 74% (74/100) female, and the mean age of all patients was 53.24 (SD 11.55) with a range of 23 to 84 years. The most common diagnosis was breast cancer (39/100, 39%). The majority of participants had completed higher education, whereas 41% (41/100) had completed secondary education and 33% (33/100) had obtained a university degree. The Distress-Thermometer indicated that the enrolled patients reported, on average, elevated and clinically relevant distress levels. The HADS scores indicated that the enrolled patients had, on average, normal scores of anxiety and depressive symptoms. Continuous app users and intervention dropouts differed in their gender, with 85% (46/85) female continuous app users versus 61% (28/61) female intervention dropouts.

**Table 1:** Baseline characteristics of all enrolled patients, continuous app users, and intervention dropouts.

Baseline characteristics		Total (N=100)	Continuous app users (n=54)	Intervention dropouts (n=46)
**Gender, n (%)**				
	Female	74 (74)	46 (85)	28 (61)
	Male	26 (26)	8 (15)	18 (39)
Age (years), mean (SD)		53.24 (11.55)	54.77 (11.27)	51.45 (11.74)
**Type of cancer, n (%)**				
	Breast cancer	39 (39)	26 (48)	13 (28)
	Colon cancer	9 (9)	4 (7)	5 (11)
	Ovarian or cervical cancer	6 (6)	6 (11)	0 (0)
	Lung cancer	6 (6)	5 (9)	1 (2)
	Others	40 (40)	13 (24.)	27 (59)
**Status of cancer treatment, n (%)**				
	Total removal	46 (46)	26 (48)	20 (44)
	Recurrence or incomplete removal	25 (25)	12 (22)	13 (28)
	Uncertain	3 (3)	2 (4)	1 (2)
	Others	26 (26)	14 (26)	12 (26)
**Highest education, n (%)**				
	Primary school	3 (3)	2 (4)	1 (2)
	Apprenticeship	22 (22)	10 (19)	12 (26)
	Secondary education	41 (41)	24 (44)	17 (37)
	University degree	33 (33)	17 (32)	16 (35)
	Unknown	1 (1)	0 (0)	0 (0)
**Setting, n (%)**				
	Setting 1^a^	17 (17)	9 (17)	8 (17)
	Setting 2^b^	83 (83)	45 (83)	38 (83)
Distress-Thermometer, mean (SD)		5.29 (2.31)	5.36 (2.47)	5.22 (2.14)
FACT-G^c^ Quality of life, mean (SD)		75.54 (13.85)	76.56 (14.08)	74.33 (13.63)
HADS^d^ anxiety, mean (SD)		6.88 (3.50)	7.17 (3.60)	6.53 (3.38)
HADS depression, mean (SD)		4.96 (2.78)	5.37 (3.05)	4.48 (2.37)

^a^Setting 1: cancer patients with a supportive Mind Body Medicine treatment.

^b^Setting 2: cancer patients without a supportive Mind Body Medicine treatment.

^c^FACT-G: Functional Assessment of Cancer Therapy-General.

^d^HADS: Hospital Anxiety Depression Scale.

### Adoption

**Figure 2:**
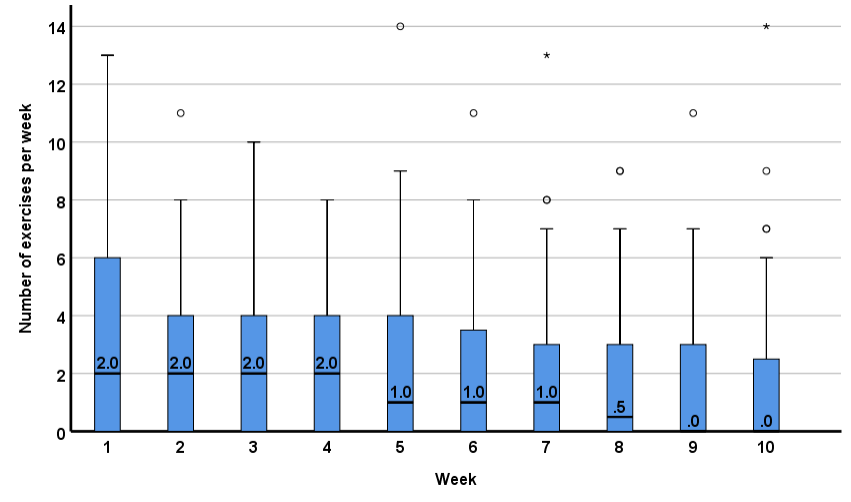
Completed app exercises by all patients who were enrolled in the study (N=100) per week (median, interquartile range).

The number of app exercises completed within the first 10 weeks of the intervention across all patients is presented in Figure 2. During the first week, the median of completed exercises was at 2 with an IQR of 0 to 6, that is, 50% of patients completed 2 or more exercises per week. Over the course of 10 weeks, the median dropped to 0 with an IQR of 0 to 2.5.

**Figure 3:**
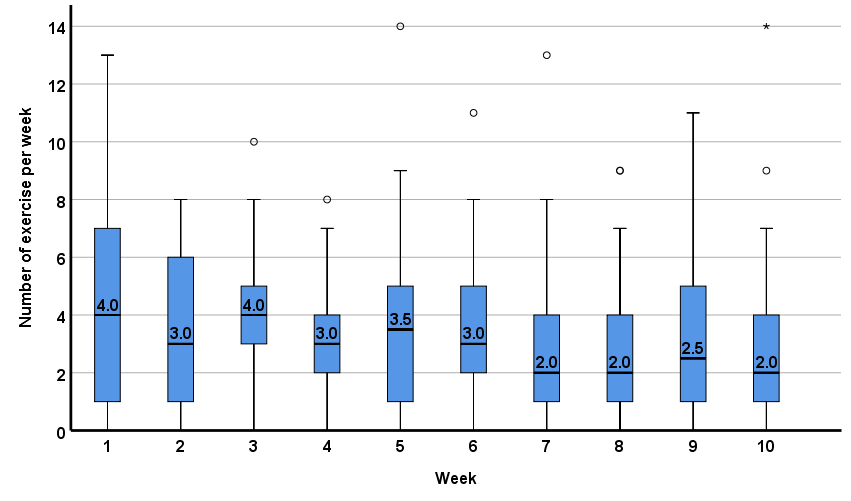
Completed app exercises by continuous app users within a 10-week app intervention (n=54) per week (median, interquartile range).

The median of app exercises completed across the first 10 weeks of the intervention for continuous app users is presented in Figure 3. During the first week, the median of completed exercises was 4 (IQR 1-7) and dropped down to a median of 2 (IQR 1-4) in week 10.

**Figure 4:**
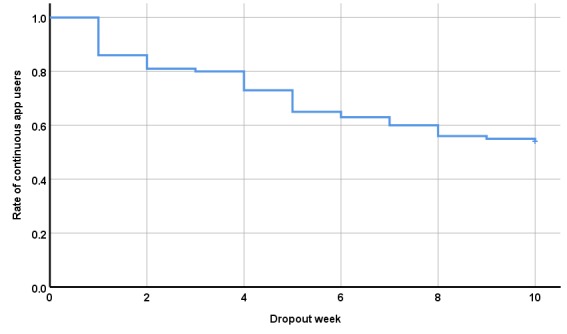
Kaplan-Meier survival curve of all enrolled patients (N=100) over 10 weeks.

The Kaplan-Meier survival curve of continuous app users is presented in Figure 4. During the first week, 14/100 (14%) patients never started or stopped using the app exercises on a regular basis and were categorized at week 1 as intervention dropouts. At the end of the intervention, 54/100 (54%) patients were using the app exercises on a regular basis, and between week 1 and week 10, the decline can be regarded as continuous without any specific sensitive weeks to drop out.

### Maintenance

**Figure 5:**
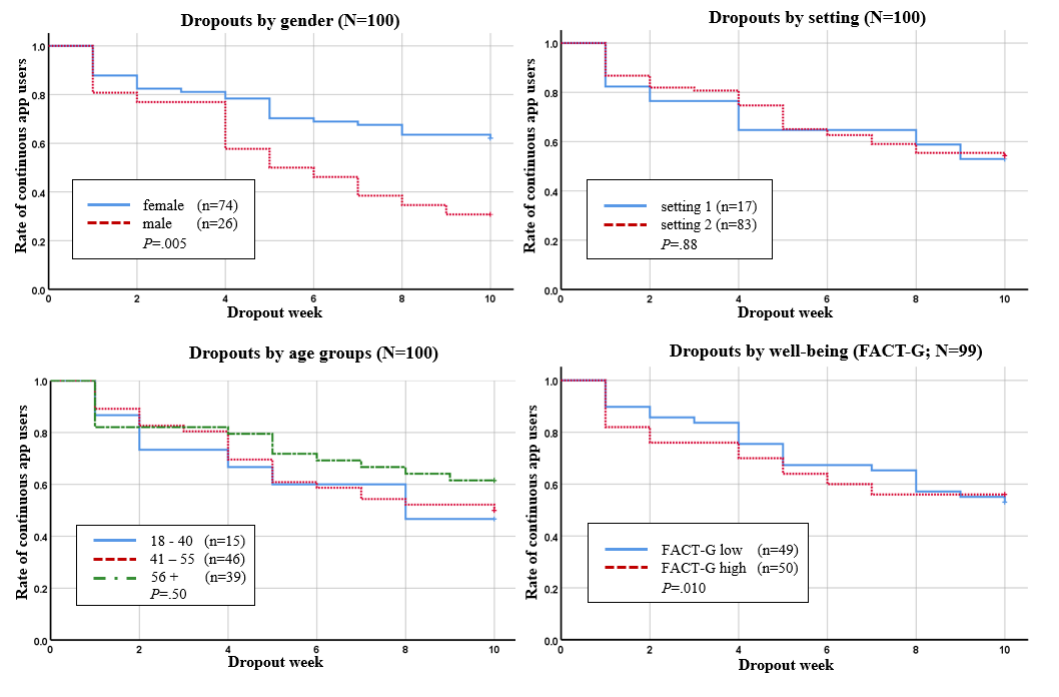
Kaplan-Meier survival curves for continuous app users by gender, setting, age groups and well-being. FACT-G: Functional Assessment of Cancer Therapy-General.

**Figure 6:**
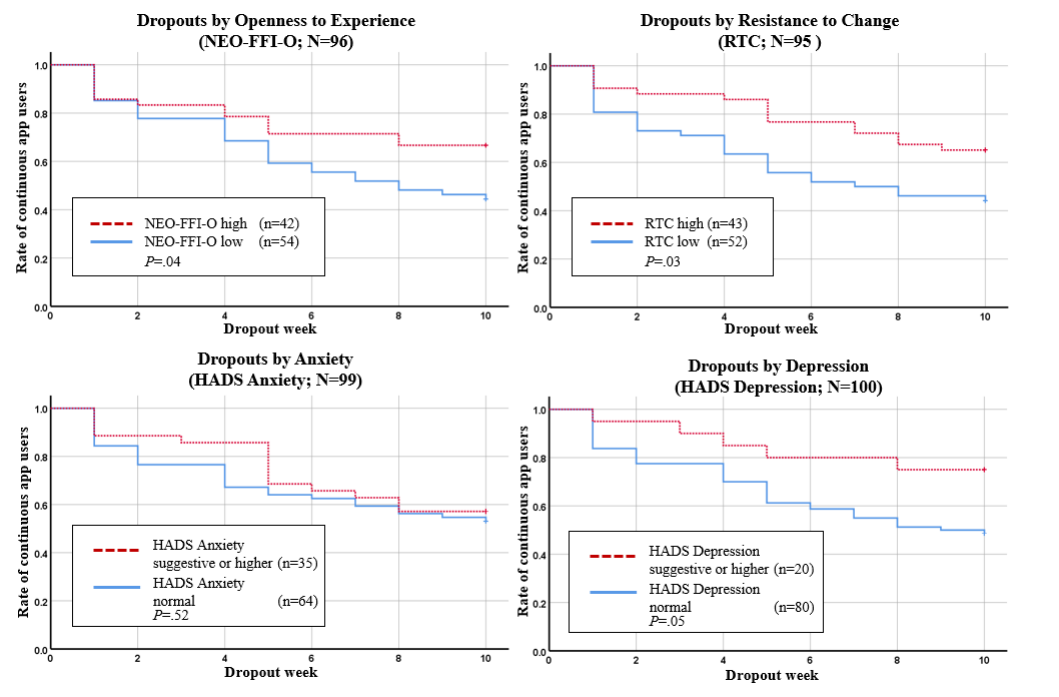
Kaplan-Meier survival curves for continuous app users by openness to experience, resistance to change, anxiety and depression. NEO-FFI-O: NEO Five-Factor Inventory - openness to experience; RTC: Resistance to Change; HADS: Hospital Anxiety Depression Scale.

The Kaplan-Meier plots for intervention dropouts by gender, setting, age groups, and well-being are presented in Figure 5. The Kaplan-Meier plots for intervention dropouts by openness to experience, resistance to change, anxiety, and depression are presented in Figure 6. Log-rank tests indicated 4 significant predictors for continuous app users, namely gender, openness to experience, resistance to change, and depression.

At week 10, 62% (46/74) of the female patients were still using the app continuously, whereas only 31% (8/26) of the male patients were using the app continuously. Therefore, females had a better adherence to use the app continuously over time than men (*P=*.005). In the high openness to experience group (NEO-FFI-O), 67% (28/42) of patients still used the app continuously through week 10. In the NEO-FFI-O low openness group, 44% (24/54) used the app continuously through week 10. Thus, patients with high openness to experience had a better adherence than patients with low openness to experience over time (*P=*.044). In patients with normal HADS depression values, only 49% (39/80) used the app exercises continuously compared with 75% (15/20) in the HADS suggestive or higher depression group (*P=*.046). In patients with high RTC, 65% (28/43) used the app exercises continuously, but in the low RTC group, only 44% (23/52)of patients used the app exercises continuously through week 10. Therefore, patients in the high RTC group had a better adherence in continuous app use (*P=*.03). For the factors setting, age groups, well-being (FACT-G), anxiety (HADS anxiety), log-rank tests did not result in significant group differences.

The 4 significant factors of the univariate log-rank test (gender, NEO-FFI-O, RTC, HADS depression) for the prediction of continuous app users went into the multivariate Cox proportional hazards regression. The multivariate analysis indicated solely gender as an independent factor for continuous app use, with an odds ratio (OR) of 2.16 (95% CI 1.09 to 4.27), with a higher chance for attrition in male cancer patients (*P=*.01). The 3 other factors (NEO-FFI-O, RTC, and HADS depression) did not contribute significantly in this analysis after controlling for gender: high openness to experience was associated with lower odds for attrition (OR 0.96; 95% CI 0.89 to 1.04; *P=*.30); high RTC with lower odds for attrition (OR 0.98, 95% CI 0.95 to 1.01; *P=*.17); more depressive symptoms with lower odds for attrition (OR 0.92, 95% CI: 0.80 to 1.03; *P=*.13).

### Qualitative Results

We invited 8 patients (2 from setting 1, 7 female, mean age 50.70 years (SD 15.06), 3 with breast cancer) to an interview, and all agreed to take part. Interviews were conducted between October 2016 and April 2017 and lasted on average 23 min. The qualitative analysis of the interviews yielded 4 themes which were as follows: (1) general feedback regarding the app, (2) suggestions for improvement, (3) personal preferences, and (4) reasons for app use and nonuse.

General feedback about the app was predominantly positive. The interviewed patients appreciated the simplicity of the app and the easy-to-use interface. One patient stated the following regarding the design:

It was great. It was very simple, very self-explanatory. You didn’t need to look around a lot and it also looked good. Yes, in any case, well designed.

Female, 35 years old

Two patients who attended an MBM course evaluated the app as a good addition to the face-to-face MBM course. The feedback about the number of exercises was mixed: Some patients regarded the implemented 3 exercises as sufficient, whereas others would welcome a larger selection of exercises. Most patients interviewed used and appreciated the reminder in the app. Some patients mentioned that they would have been less compliant without the reminders and, therefore, perceived the reminder as helpful for a continuous app use. For instance, 1 patient stated the following:

Yes, [the reminder function] was very good. A couple of times this was very good. I would have forgotten it a couple of times, if I wouldn’t have had this reminder.

Female, 63 years old

Patients offered various suggestions for improvement. Several patients mentioned that they would like exercises with background music. One patient explained it as follows:

I think it is also precisely the high art of meditation or relaxation that you can relax as much as possible while not falling asleep. Some need chimes, while others need absolute silence to be able to do this. And I realize that when it is absolutely silent, either I fall asleep or I start to contemplate. When I have some music or chimes, it works better for me personally.

Female, 42 years old

As stated above, some patients also would welcome a larger variety of exercises (eg, autogenic training) or variations in the duration of exercises. Another patient stated also that the recordings of the exercises were too clean (ie, no noises from breathing), as the exercises were recorded in a studio. This led to the patient being startled when the narrator continued with the instruction after a moment of silence. One patient mentioned she did not set up the reminder during the first time she used the app and later forgot about the reminder function. Therefore, this patient suggested that the reminder function could be placed more prominently in the app instead of the options menu. The interviewer also inquired if the patients would appreciate a feedback system in the form of exercise statistics. The majority of interviewed patients had the opinion that such a feature would not be helpful. Some patients stated that statistics might even be stressful, as it might lead to a guilty conscience if the patient is not using the exercises as often as planned. One patient suggested that statistics might be added to the app as an optional feature. Only 2 patients thought that such a feature might be helpful.

The third topic that emerged from the qualitative analysis was personal preferences. Most interviewed patients mentioned that they developed some form of preference regarding the app use (eg, preference for a specific exercise, gender of narrator, time of day when using the exercises) during the intervention while they were trying out what suits them best. One patient stated the following:

Right at the beginning I tried [to do the exercises] before I went to bed. But I’m not a fan of having my cellphone, when I fall asleep, next to my head for the entire night. For this reason I changed it to lunchtime.

Female, 31 years old

The fourth topic that emerged from the qualitative analysis involved reasons for app use and nonuse. As a reason for using the app, patients mentioned that the exercises were beneficial and helped them to relax. One patient stated the following:

[The app exercises] have been good for me. I will continue to do my exercises. […] I believe I benefit [from the exercises]. It also makes you happy.

Female, 63 years old

As reasons for nonuse, 3 patients mentioned that they had previous experience with meditation or relaxation exercises. Therefore, these patients were already used to exercise routines, which differed from the instructions or the manner in general of the app exercises. One of these patients mentioned that she had learned and was used to silent meditation, and therefore the guided exercises in the app were more distracting than helpful to her. Another patient mentioned that she had experience in guided meditation and relaxation exercises, which differed linguistically and in form of conduct compared with the app exercises. This patient mentioned that she was unable to get used to these new exercises and was repeatedly comparing the app exercises with the already known exercises. Therefore, this patient could not relax as intended during the app exercises. A third patient mentioned that she was used to exercises with more guidance and described her experience with the app as follows:

Maybe because [the instructions in the app exercises] were different from what I was used to do by myself, where [the exercise] was guided the entire time. […] I did consider it more bothersome that…[…] your thoughts drift away because you get the feeling that [the exercise] should continue.

Female, 49 years old

As a further reason for nonuse, 1 patient mentioned that she was distracted by the choice of words and expressions in the app exercises. This patient mentioned that she had studied linguistics and had learned to closely scrutinize language. This caused her to be distracted during the app exercises, which is why she stopped using the app. Another patient mentioned that she suffered from cancer-related fatigue and that she was not able to complete an exercise when she was unduly fatigued. Furthermore, 1 patient stated he had technical problems with his smartphone and therefore was not able to use the app during the entire 10 weeks.

## Discussion

### Summary of Findings

mHealth interventions with the aim of reducing distress in cancer patients seem promising due to easy access and potential positive effects for patients. To our knowledge, this is one of the first studies looking in detail at characteristics of users, adherence rates, and possible predictors for adherence in a mindfulness and relaxation mHealth study for cancer patients. This feasibility study showed that adherence to the mHealth intervention during the first 10 weeks was acceptable, with 54% of patients still using the app regularly in week 10 with a median of completed exercises ranging from 2 to 4 per week. Therefore, our study does not confirm the concern that adherence in mHealth interventions is in general poor, which would limit treatment implementation. The adherence of our patients is also comparable with recent research on adherence to e- and mHealth interventions for cancer patients [32,33]. A study by Beatty el al [32] reported that 60% of cancer patients completed 4 or more modules of an eHealth intervention with 6 modules, which aimed at reducing distress in cancer patients. A mindfulness app study for cancer patients and caregivers [33] reported that 71% of the participants practiced with the app on more than half of the days throughout 8 weeks.

The uptake of our intervention was good, with 117 screened and eligible patients, of whom 100 patients returned the informed consent form. In addition, 74%, mainly female, patients enrolled in this study, which is consistent with characteristics of mHealth users in other studies with 84% female patients [47] and 54% female patients [48]. The mean age of participating patients was 53 years; this is comparable with other face-to-face mindfulness and relaxation interventions [49] or Web-based interventions for cancer patients [32]. The interviews showed that the patients were satisfied with the app in general. However, several and sometimes contradictory suggestions were made for improving the app, such as less versus more guidance in the exercises and larger variety in exercises versus the notion that 3 exercises are sufficient.

### Predictors for Adherence

Of a total of 8 investigated predictors for continuous app use, 4 turned out to be statistically significant. The strongest predictor was gender, with higher adherence in female cancer patients. Beyond the higher interest of female cancer patients to participate in this mindfulness and relaxation mHealth study, they were also more adherent after starting with the exercises. This result is in line with a study by Ruland et al [50], in which an analysis of use patterns in an eHealth intervention to support cancer patients’ illness management revealed that female patients used the system almost twice as often as male patients. However, a study by Duman-Lubberding et al [51] investigated the feasibility of a Web-based self-management app and did not find a gender difference in adherence. Therefore, it seems likely that the type of intervention (eg, relaxation and mindfulness meditation) might be relevant for gender differences in adherence, which is also in line with studies about the use of complementary and alternative medicine, where users tend to be more often female [52,53].

A second predictor for continuous app use was the personality trait openness to experience, whereby higher openness to experience predicted more continuous app use. This result fits with previous research, which has shown that openness to experience predicts the use of complementary and alternative medicine, including mindfulness and relaxation [54,55]. Our study confirms that higher openness to experience still predicts the adherence to a mindfulness and relaxation intervention, even if the intervention is delivered through an app.

A third predictor for continuous app use was a higher score in resistance to change. This finding is contrary to our hypothesis, as we assumed that higher resistance to change would be associated with less adherence as the intervention promotes a new health behavior. However, our results indicate the opposite. When a patient has decided to follow a new exercise routine (ie, mindfulness and relaxation mHealth intervention), a higher resistance to change actually promotes continuous app use. To our knowledge, the Resistance to Change Scale had not previously been used to predict adherence to mHealth interventions for cancer patients. However, a study conducted in China by Deng et al [56] showed that resistance to change is negatively related to the intention to use mHealth services. Another study showed that resistance to change is negatively related to perceived usefulness of mHealth in elderly people in China [57]. Therefore, on the one hand, resistance to change might be a barrier for the uptake of an mHealth intervention, but on the other, it might be supportive in adhering to a new commitment, such as the regular use of a mindfulness and relaxation app.

A fourth predictor for continuous app use was higher depressive symptoms. This finding is surprising, as depressive symptoms are associated with decreased motivation and reduced activity [58]. In line with these corollaries, a study investigating a mindfulness-based cancer recovery program [49] reported a negative correlation of depressive symptoms and practicing time of yoga at home. Another study [59] reported that moderate to severe depressive symptoms predicted lower adherence to adjuvant cancer therapies. However, a study by Børøsund et al [60] found that high levels of depression were associated with high use of components of a Web-based illness management program in breast cancer patients. As depressed patients are oftentimes troubled with motivational deficits and face difficulties to stay active, the development of effective interventions with a good adherence in depressed patients is highly relevant. Our study indicates that mindfulness and relaxation mHealth interventions seem a feasible tool as supportive interventions for cancer patients with elevated depressive symptoms. This finding might also indicate that some patients adhere better to mindfulness and relaxation (ie, patients with higher depressive symptoms), whereas other patient groups with lower levels of distress are not in need of such interventions or might prefer other intervention types.

### Limitations

This study has some limitations. First, the number of potentially interested patients for this intervention could not be assessed. Therefore, we were not able to calculate the rate of the total number of eligible cancer patients compared with the number of cancer patients with interest in a mindfulness and relaxation mHealth intervention. Second, for our definition of continuous app, we had no empirical data because the necessary dose for clinically significant improvements is still unclear for this kind of mHealth intervention. Instead, we opted for a clinical and rational justification, in which the term “continuous” use was operationalized as an at least weekly use of 1 or more app exercises. Third, the use of generated categories for age and the median split for other variables as predictors can be challenged. For age, we chose 3 age categories that represent patients of younger (18-40), middle (41-55), and older (56 plus) age. The use of median split variables has been critically discussed in the literature (see eg, Iacobucci et al [61]), with a major critique being the loss of information. In our case, the loss of information can be justified with the illustrative capacity of Kaplan-Meier survival curves and the following use of a multivariate Cox proportional hazards regression. Fourth, as the sample size was an outcome in itself, we did not perform an a priori sample size calculation. With a sample size (N) of 100, we had a power of 0.63 in the Cox proportional hazards regression for the main effect (OR 2.16) of gender as a predictor. For a power of 0.8, a sample size (N) of 150 would be necessary.

### Conclusions and Future Research

The acceptable adherence to the intervention and the generally positive feedback by patients indicate that this app intervention is feasible. Suggestions for improvement by patients indicate that patients’ needs are heterogeneous, which should be taken into account when developing other mHealth interventions. Due to the acceptable adherence and positive feedback by cancer patients, mindfulness and relaxation mHealth interventions might be promising supportive interventions, also for cancer patients with elevated depressive symptoms.

To further prove the importance of mindfulness and relaxation mHealth interventions for cancer patients, future research needs to investigate their effectiveness. As the dose potentially influences the effectiveness of mindfulness and relaxation interventions, future research should also look into dose-response relationships between the time spent exercising with the app and health outcomes. Knowledge of such a dose-response relationship could be of use to guide subsequent studies regarding intervention duration and practice recommendation for patients. This study suggests that variability across patients in weekly app use is large. About half of the patients used the app exercises continuously over 10 weeks and therefore adhered to the intervention. These interindividual differences in the use of app exercises underline the importance to take adherence into account when analyzing effectiveness data. Furthermore, these interindividual differences on adherence bring up the question if mindfulness- and relaxation-based mHealth interventions are better suited for specific patient groups (eg females, patients with higher depressive symptoms). In turn, male patients or patients with less distress might not be in need of such interventions or might require additional motivational interventions.

## Appendix

Multimedia Appendix 1 Screenshots of the CanRelax App exercises.

Multimedia Appendix 2 Screenshots of the CanRelax App notification feature.

Multimedia Appendix 3 Interview guideline.

## Abbreviations

CRF: case report form
FACT-G: Functional Assessment of Cancer Therapy-General
FMI: Freiburg Mindfulness Inventory
FoP-Q-SF: Fear of Progression Questionnaire - Short Form
HADS: Hospital Anxiety Depression Scale
ICI: Institute for Complementary and Integrative Medicine, University Hospital Zurich
IQR: interquartile range
MBM: Mind Body Medicine
mHealth: mobile health
NEO-FFI-O: NEO Five-Factor Inventory - openness to experience
OR: odds ratio
PROMIS 29: Patient Reported Outcomes Measurement Information System 29
RTC: Resistance to Change
