# Supplementary figures and images for: Adherence to a Mindfulness and Relaxation Self-Care App for Cancer Patients: Mixed-Methods Feasibility Study
Source: JMIR Mhealth Uhealth. 2018 Dec 6;6(12):e11271. doi: 10.2196/11271 (PMC6302233; doi:10.2196/11271)

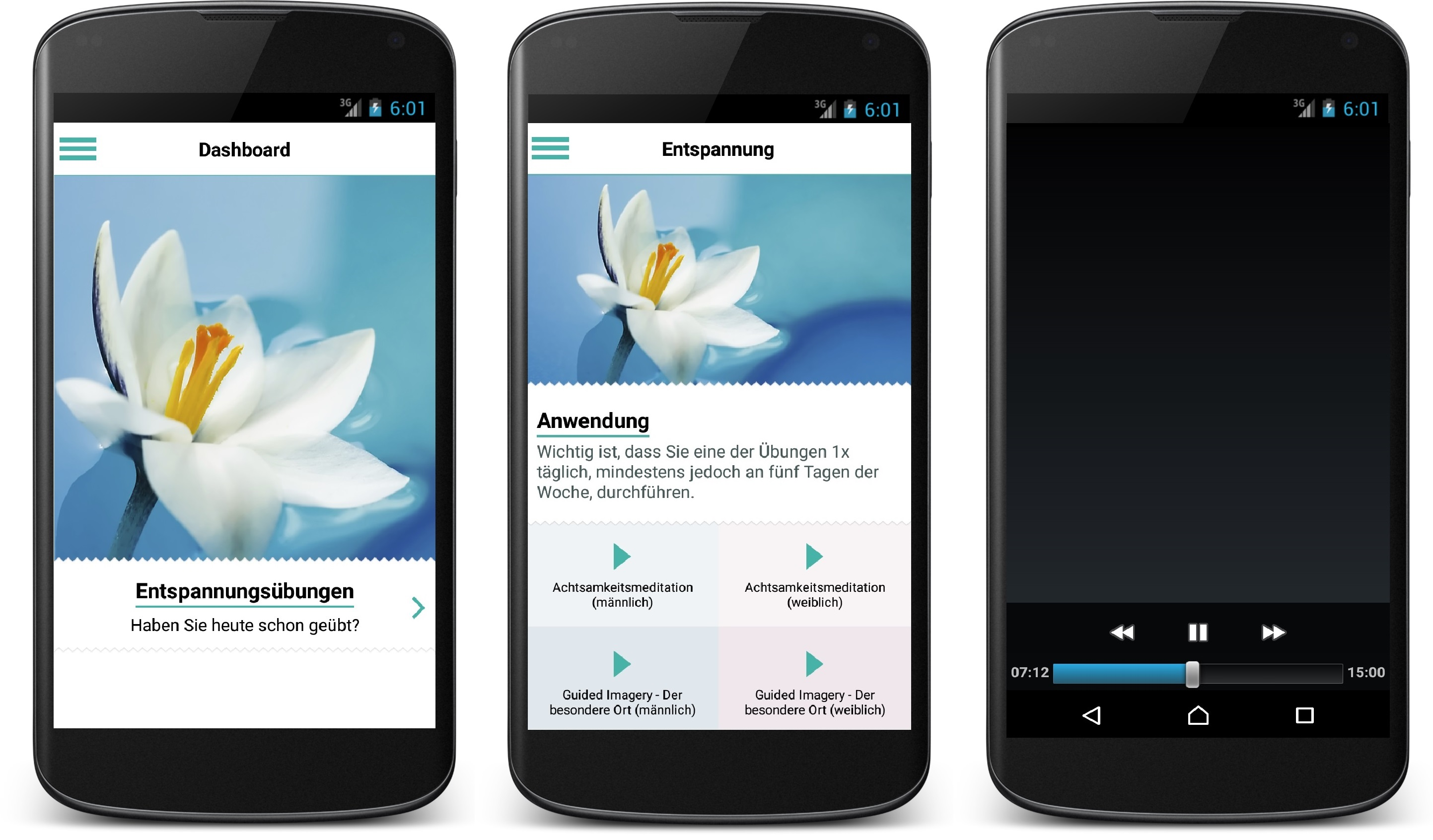

Supplement: Multimedia Appendix 1 [file mhealth_v6i12e11271_app1.png]

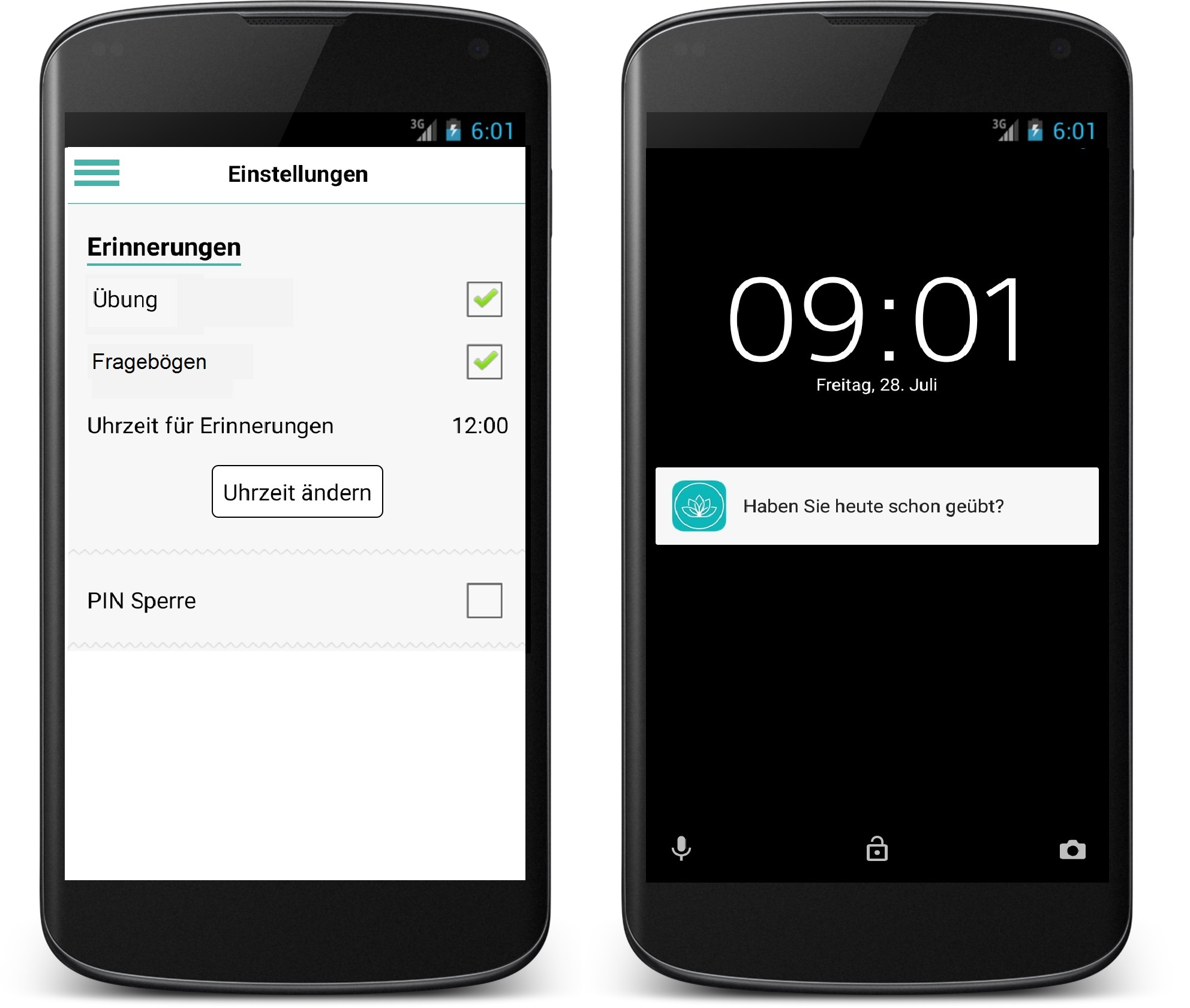

Supplement: Multimedia Appendix 2 [file mhealth_v6i12e11271_app2.png]
