# Supplementary material for: Adherence to a Mindfulness and Relaxation Self-Care App for Cancer Patients: Mixed-Methods Feasibility Study
Source: JMIR Mhealth Uhealth. 2018 Dec 6;6(12):e11271. doi: 10.2196/11271 (PMC6302233; doi:10.2196/11271)
Supplement: Multimedia Appendix 3 [file mhealth_v6i12e11271_app3.pdf]

Interview questions

Before the interview

Before the recording begins, the patients are informed about the following points and they received a consent form for the interview:

- Introduction of each other
- Duration of the interview
- Content of the interview
- Type of the interview
- Handling of the collected data
- Clarification of open questions

Opening question

- What is your general impression of the app?

Questions regarding the evaluation of the app

- How satisfied are you with the app?
- How satisfied are you with the exercises of the app?
- How satisfied are you with the usability of the app?

Questions regarding the use of the app

- How did you use the app?
- When and in which situations did you use or not use the app?
- At the beginning of the study, did you have any idea on how you would like to use the app?

Questions regarding the evaluation of the app and suggestions for improvement

- How do you think the app can be improved?
- What should be retained in the app?
- What should be improved in the app?
- In such an app, do you wish more exercises or less exercises, or are the three available exercises just right?
- Would you like getting a statistical feedback, where you can keep track about how often you have practiced per week?

Closure of the interview

- Is there anything else that you would like to add or anything we have not talked about, but that you find important to mention?

Thank you for the interview.
